# Healthcare utilization and costs of singaporean youth with symptoms of depression and anxiety: results from a 2022 web panel

**DOI:** 10.1186/s13034-023-00604-z

**Published:** 2023-05-11

**Authors:** Parth Chodavadia, Irene Teo, Daniel Poremski, Daniel Shuen Shung Fung, Eric A. Finkelstein

**Affiliations:** 1 Duke University School of Medicine, 8 Searle Center Drive, Durham, NC 27705 USA; 2 Lien Centre for Palliative Care, Duke-NUS Medical School, 8 College Rd, Singapore, 169857 Singapore; 3 Institute of Mental Health, Singapore, Singapore; 4 Department of Developmental Psychiatry, Institute of Mental Health, Singapore, Singapore; 5 Duke-NUS Medical School, 8 College Rd, Singapore, 169857 Singapore

## Abstract

**Background:**

There is evidence that the prevalence of depression and anxiety among youth is increasing and that these factors contribute to high healthcare costs and poor school performance. The goal of this study is to provide up-to-date estimates of the prevalence and economic burden of depression and anxiety among youth in Singapore.

**Methods:**

Using an existing web panel, 991 parents filled out the PHQ-4 screener on behalf of 1,515 youth. 104 of these parents whose children (ages 4 to 21) had symptoms consistent with depression or anxiety filled out a full survey with questions on mental health symptoms, school absences, school performance, and healthcare utilization. The survey was fielded between April and June 2022. Publicly available prices were used to estimate the cost associated with the observed rates of health service use.

**Findings:**

Based on parental responses, 11.7% (95% CI:10.2 − 13.5%) of youth had symptoms consistent with depression and 12.8% (95% CI:11.2 − 14.6%) had symptoms consistent with anxiety. In total, 16.2% (95% CI:14.5 − 18.3%) were reported to have symptoms consistent with at least one of these conditions. These youths missed an average of 190 (95% CI: 126–254) hours of school per year due to their mental health conditions and parents reported that school and daily activities performance was significantly degraded. Per capita annual healthcare costs averaged S$10,250 (95% CI: 7,150–13,350), with 64% of youth receiving emergency or inpatient services. In aggregate, annual costs associated with these conditions were estimated to be S$1.2 billion (95% CI:S$1.1bn – S$1.4bn).

**Interpretation:**

Even with significant potential for underreporting, these results reveal concerning rates of Singaporean youth with symptoms consistent with depression or anxiety, many of whom remain untreated. Results also reveal the short-term economic burden caused by these symptoms and hint at longer-term consequences resulting from poor school performance. This study should represent a call to action for Singapore to address poor mental health among youth.

**Supplementary Information:**

The online version contains supplementary material available at 10.1186/s13034-023-00604-z.

## Introduction

Depression and anxiety are the leading causes of morbidity among youth worldwide and collectively contribute to more than ten million disability-adjusted life years annually [1]. This results, in part, because these two conditions are responsible for half of all suicides among youth. Youth with depression or anxiety are also more likely to suffer from additional comorbid behavioral problems such as attention deficit hyper-activity disorder and conduct disorder, have decreased school performance, and are more likely to engage in risky behaviors such as risky sexual behaviors and drug and alcohol abuse [2, 3]. For these reasons, they are also responsible for a disproportionate share of annual medical expenditures [4–6]. Longer term, youth with these conditions can expect 30% lower lifetime earnings due, in part, due to lower academic achievement, and are less likely to have stable family relationships [7, 8].

Even before the COVID-19 pandemic, rates of depression and anxiety among youth were increasing in high-income countries. Several countries, including the United States and Finland, had shown a doubling in prevalence in the decade leading up to the pandemic [9, 10]. Increased social media use, combined with prolonged school closures, social isolation, and general household stress associated with the pandemic has further exacerbated the prevalence of depression and anxiety among youth [11]. A recent systematic review found that the prevalence of these conditions had more than doubled to 25% and 21% respectively in the first year of the pandemic [11].

Policymakers in Singapore, the country of focus of this effort, rely on timely information of the prevalence and economic burden of select conditions in efforts to prioritize prevention and treatment initiatives. Although estimates from other countries are available, they may not generalize to Singapore due to myriad factors, including cultural difference and differences in health seeking behavior. Prior to the COVID19 pandemic, although no economic burden data exist, Magiati et al. reported that 9.3% and 16.9% of 8–12 year-old Singaporean children had clinically elevated symptoms of anxiety and depression, respectively, in 2015 based on a representative household survey of Singaporean children [12]. At the height of COVID-19 pandemic from April to June of 2020, home-based learning was instituted across all educational institutions [13]. Although schools were reopened in June 2020, school activities remained restricted throughout 2020 and 2021 [14]. Given school closures and other COVID-era restrictions in the city-state, [15] and increases in social media use and other risk factors, rates of depression and anxiety have likely increased. Currently, no updated information is available for depression and anxiety prevalence among youth and the corresponding economic burden.

As was done by Magiati et al., the gold-standard approach for generating this information is through in-person school- or household-based surveys [12]. However, these are time-consuming, costly, and difficult to implement. An alternative, convenient and low cost approach, which we take in this study, is to rely on parent reporting of youth prevalence and economic burden via a web panel. We use an existing web panel to estimate the prevalence of depression and anxiety symptoms among Singaporean youth based on parent report and to determine the extent to which these symptoms increase school absences, reduce school performance, hamper daily activities, and increase healthcare utilization/costs. We hypothesize that these symptoms will lead to increases in healthcare utilization and costs and reductions in school performance and performance in regular activities (outside of school). Although we focus on Singapore, this approach can be easily replicated in countries worldwide.

## Methods

### Participants

A cross-sectional online survey was administered in English, the official language in Singapore, to adult residents (age > 21) who are members of a national web panel curated by Kantar Profiles Division. This cross-sectional survey was part of a broader exercise to quantify the prevalence and economic burden of depression and anxiety in both adults and children. Participants were recruited between April 20, 2022 and June 1, 2022 through email invitations and asked to fill out a screener questionnaire to identify symptoms consistent with depression or anxiety disorder among all household members as indicated by the PHQ-4 [16] (Supplementary Appendix A). Participants were asked to fill out the screener for themselves and for all members living in their household. If the main respondent did not have symptoms consistent with depression or anxiety and at least one youth between the ages of 4 and 21 in their household did, then the main respondent filled out a detailed questionnaire on behalf of the oldest child. This manuscript focuses exclusively on results for youth. IRB approval was obtained prior to fielding the survey (IRB# 2021 − 836).

### Setting

The first case of COVID-19 in Singapore was detected in January 2020 [17, 18]. Community cases swelled in March 2020 and a quarantine, termed locally a “circuit breaker”, was imposed on April 7th 2020 for two months where public movement was limited to essential activities [19]. Pre-schools, schools, and universities introduced home-based learning at this time [20, 21]. In the following months schools reopened but social gatherings were limited to control the spread of subsequent outbreaks and mask mandates were in effect in public spaces. In January 2021, vaccination programmes began and by December 2021, 80% of the population was fully vaccinated. By March 2022, international travel restrictions were lifted and by May 2022, almost all pandemic-related infection control measures were lifted. However, indoor mask mandates remained in effect to February 2023, when Singapore considered the virus endemic [22].

### Measures

The PHQ-4 is the combination of the PHQ-2 and GAD-2, which are validated two-item, two-week recall period, ultra-brief screeners shown to have high sensitivity and specificity for identifying depression and anxiety disorder [16, 23−25]. The PHQ-4 includes four questions about the frequency of which respondents feel: 1 - Nervous, anxious or on edge; 2 - Not being able to stop or control worrying; 3 - Down, depressed, or hopeless; and 4 - Little interest or pleasure in doing things. Responses were recorded on a 4-point Likert scale, including: 1 - Not at all, 2 - Several days, 3 - More than half the days, 4 - Nearly every day. Although the PHQ-4 is not a diagnostic tool for depression or anxiety, its brevity allows for rapid identification of individuals who have symptoms consistent with these conditions. Moreover, the PHQ-2 and GAD-2 are validated against the broader PHQ-9 and GAD-7 across multiple countries in adolescents [26–29]. At the cutoff scores of 3 or greater, PHQ-2 had a sensitivity of 74% and specificity of 75% for detecting youth who met Diagnostic and Statistical Manual of Mental Disorders [26]. GAD-2, at cutoff scores of 3 or greater, had specificity and sensitivity of 0.84–0.87 and 0.93–0.95 respectively against the GAD-7 [30].

Parent proxies were asked to complete the full survey for their oldest eligible child only if they themselves did not have symptoms of depression or anxiety. This approach limited survey fatigue as parents had to fill the survey out only one time regardless of how many individuals qualified in the household. Parent proxy-reported psychosocial measures have a high degree of reliability and validity to patient-reported outcomes in pediatric populations [31–33]. The full survey (available in Supplementary Appendix A and B) included the following domains: Mental Healthcare Utilization, Productivity Losses from Absenteeism and Presenteeism, Quality of Life Measures, and Preferences for Peer Support. The full survey also included socioeconomic questions about the youth (e.g. age, gender, education level, and about the primary caregivers (e.g. employment status and monthly income of caregivers).

### Prevalence analysis

To estimate prevalence among youth and to determine eligibility for the full survey instrument, panelists were asked to report the ages of all youth in the household and complete the PHQ-4 for each child. A child was assumed to have symptoms consistent with anxiety if they scored 3 or higher on the anxiety sub-scale (sum of items 1 and 2) [34]. Similarly, a child was assumed to have symptoms consistent with depression if they scored 3 or higher on the depression sub-scale (sum of items 3 and 4). Previous research has established that a score of 3-or-greater on the Depression and Anxiety subscales represent a reasonable cut-point for identifying potential cases of major depression and generalized anxiety respectively [34]. Prevalence rates were calculated by dividing the number of children who have symptoms consistent with anxiety or depression by the total number of reported children across all households in the screener. We present overall estimates for each condition and report the percentage ‘never being told by a healthcare professional that the child has depression or anxiety’, and thus who likely remain untreated. All results are based on the parent proxy responses.

### Healthcare resource utilization analysis

Before conducting any analyses, data was cleaned and suspicious responses were re-coded as missing. We recoded 17 (1.7%) and 38 (3.8%) responses from healthcare resource utilization and school absenteeism, respectively, to missing. This is due to respondents reporting some form of healthcare visits or missed school hours despite indicating “No healthcare utilization” and “No absence from school”. Details on data cleaning are available in Supplementary Appendix C. Children who met the threshold for PHQ-4 and had full survey data were included in the healthcare resource utilization analysis. To quantify healthcare utilization attributable to depression or anxiety, participants were asked about the frequency of physician and outpatient visits (including tele-visits), medications, and alternative therapies (e.g., acupuncture, reflexology) for their eligible child. For these questions, the recall period was three months. Other questions focused on diagnostic tests, emergency department visits, and number and duration of hospitalizations. For these we used a recall period of twelve months as these episodes are less frequent and easier to remember [35]. To monetize healthcare utilization, unit costs were applied to each type of service based on unsubsidized costs collected through publicly available sources. Full breakdown of unit costs and assumptions are available in Supplementary Table 1. Per capita healthcare cost estimates were taken by averaging across responses. Total cost estimates were generated by multiplying the total youth population (between ages 4 and 21) from the Singapore Department of Statistics, by our estimated prevalence rates and then by the per capita cost estimates.

### School absenteeism and performance

School absenteeism was determined by multiplying the estimates of weekly hours missed from school due to depression and anxiety by the number of weeks in a school year (i.e., 40 weeks). School performance was captured by a question asking parent proxies the degree to which the youth’s depression or anxiety symptoms affected performance at school on a scale of 0–10 with 0 being “no symptoms or symptoms had no effect on my child’s school performance” and 10 being “symptoms completely prevented my child from attending school”. Similarly, performance of regular daily activities was captured by a question asking parent proxies the degree to which the youth’s depression or anxiety symptoms affected their ability to do his or her regular daily activities (other than school performance) on a scale of 0–10 with 0 being “no symptoms or symptoms had no effect on my child’s regular daily activities” and 10 being “symptoms completely prevented my child from his or her regular daily activities”.

### Statistical analysis

All costs are reported in 2022 Singapore dollars (S$) and also reported in 2022 USD^1^. 95% confidence intervals (CI) for prevalence and mean results are estimated using standard approaches. To obtain the 95% confidence interval for the total cost estimate, which is a function of both prevalence and unit costs, we ran 1,000 Monte Carlo simulations. In each run, we drew a prevalence proportion (*p*_*i*_) from a beta distribution centered at the sample prevalence proportion and standard deviation based on the standard error of the prevalence estimate. We then calculated total number of youth with symptoms consistent with depression or anxiety (*k*_*i*_) as the product of *p*_*i*_ and the Singapore population aged 4 to 21. We then drew *k*_*i*_ observations with replacement from the distribution of costs in our sample and summed to generate the total cost for each iteration. 95% CI interval was calculated based on the 2.5th and 97.5th percentile of the total costs obtained from the 1,000 simulations. Given results showing that depression and anxiety symptoms co-occur in the vast majority of cases, we present burden estimates for depression and anxiety combined. All analyses were conducted in Stata/SE 17.0 (College Station, Texas, United States).

## Results

### Prevalence of depression and anxiety symptoms

In total, 991 panelists filled out the PHQ-4 depression/anxiety screener on behalf of 1,515 youth living in these households. Out of these youth, 118 (11.7%, 95% CI: 10.2 − 13.5%) had symptoms consistent with depression and 194 (12.8%, 95% CI: 11.2 − 14.6%) had symptoms consistent with anxiety. Based on the PHQ-4 responses, 64.4% of youth with symptoms consistent with depression also had symptoms consistent with anxiety and 70.2% of youth with symptoms consistent with anxiety also had symptoms consistent with depression. In total, 247 (16.2%, 95% CI: 14.5 − 18.3%) were reported to have symptoms consistent with at least one of these conditions yet, based on parent report, 84.8% did not have a formal diagnosis of either condition.

### Demographic and clinical characteristics

Among the children on behalf of whom the parents filled out the full screener (n = 104), the mean depression subscale-score was 3.93 (SD 0.97) (out of 6). Among children with symptoms consistent with anxiety, the mean anxiety subscale-score was 3.90 (SD 1.01) (out of 6). The mean age of the sample (n = 104) was 10.6 (SD 5.2) years; 41.4% were female; 29.8% were aged between 4 and 6; 26.9% were aged 14 or above; 43.3% were enrolled in primary school and 31.7% were enrolled in secondary school. In line with population estimates, 78.9% were Chinese. 74.0% of the children come from households with incomes greater than S$5,000 a month. Other characteristics of the sample can be found in Table 1.

**Table 1:** Baseline characteristics by PHQ-4 status (N = 104)

*Demographic Characteristics*	Total (N = 104)
**Mean Age (SD)**	10.6 (5.2)
**Female, n (%)**	42 (40.4%)
**Chinese, n (%)**	81 (77.9%)
Education Level, n (%) **	
No Formal Education	8 (7.7%)
Primary School (≤ 6 years)	45 (43.3%)
Secondary School (≤ 10 years)	33 (31.7%)
Vocational / Junior College (> 10 years)	18 (17.3%)
**Parent Proxy Employment Status, n (%)**	
Employed	43 (41.4%)
Not Employed	10 (9.6%)
Not Applicable or Missing	51 (49.0%)
**Caregiver Monthly Income, n (%)**	
S$ 0 to S$ 1,999	9 (8.7%)
S$ 2,000 to S$ 4,999	28 (26.9%)
S$ 5,000 to S$ 9,999	22 (21.1%)
S$ 10,000+	19 (18.3%)
Not Reported or Missing	26 (25.0%)
Primary Caregiver at Home ***, n (%)	
Respondent	35 (34.0%)
Spouse of Respondent or Co-Parent	41 (40.0%)
Other Family Member of Friend	13 (13.0%)
Helper / Paid Caregiver	4 (4.0%)
***Clinical Characteristics***	
**Mean PHQ-4 Score**	
Mean Total Score (SD)	6.5 (2.3)
Mean Depression Sub-Score (Among Depressed) (SD)	3.9 (1.0)
Mean Anxiety Sub-Score (Among Anxiety) (SD)	3.9 (1.0)

* Columns for each characteristic may not sum up to 100 due to rounding

** Children with no formal education were too young to be enrolled in an educational programme (4–5 years old)

*** Respondents could select more than one option for primary caregiver at home taking care of youth

### Annual healthcare utilization

Over the past year, 63% of children with symptoms consistent with depression or anxiety visited the ED and 54% were admitted to the hospital. The average number of ED visits per year was 1.0 (95% CI: 0.7–1.3), and the average length of stay across all admission was 2.9 days (95% CI: 1.4–4.4). In total, 77% of respondents reported that their child obtained healthcare to treat their mental health condition over the past three months and 62% reported some medication use. 37% reported using a daily anti-depressant or anti-anxiety medication; 72% reported in-person physician consults, and 62% reported tele-consults. Among children who saw a mental healthcare provider, psychiatrists and psychologists were most frequent. The average number of visits to psychiatrists and psychologists were 1.3 (95% CI: 0.4–2.2) and 1.2 (95% CI: 0.3–2.1) visits per year respectively. A detailed breakdown of healthcare resource utilization is available in Table 2.

**Table 2:** Summary characteristics of healthcare utilization among children and youth who screened positive for MDD or GAD

**Total (N = 104)**		**Any utilization**		
**Recall Period**	**Healthcare Cost**	**n (%)**	**Total Frequency of Use**	**Mean Utilization (95% CI)**
N/A	**No Healthcare Utilization**	**22 (21%)**	**N/A**	**N/A**
3 Months	Medication Use	65 (63%)	N/A	N/A
	Daily Medication	39 (37%)	39	N/A
	As Needed Medication	51 (49%)	51	N/A
	Insomnia Medication	19 (18%)	19	N/A
	Medication Not Listed	0 (0%)	0	N/A
	Other Medication	0 (0%)	0	N/A
3 Months	In-Person Physician Consultations	74 (72%)	N/A	N/A
	Polyclinic	62 (60%)	192	1.8 (0.7–3.0)
	Private GP	45 (43%)	120	1.2 (0.5–1.8)
	Psychiatrist	41 (39%)	135	1.3 (0.4–2.2)
	Psychologist	37 (36%)	129	1.2 (0.3–2.1)
	Social Worker	31 (30%)	87	0.8 (0.3–1.4)
	Life Coach	26 (25%)	73	0.7 (0.2–1.2)
	Other Visit Status	21 (20%)	35	0.3 (0.2–0.5)
3 Months	Tele-Physician Consults	64 (62%)	N/A	N/A
	Polyclinic	51 (49%)	123	1.2 (0.8–1.5)
	Private GP	34 (33%)	117	1.1 (0.4–1.8)
	Psychiatrist	33 (32%)	79	0.8 (0.4–1.1)
	Psychologist	32 (31%)	64	0.6 (0.3–0.9)
	Social Worker	28 (27%)	61	0.6 (0.3–0.9)
	Life Coach	21 (20%)	51	0.5 (0.2–0.8)
	Other Visit Status	21 (20%)	36	0.3 (0.2–0.5)
1 Year	Hospitalization Status	65 (63%)	N/A	N/A
	ED Visits (Count)	61 (59%)	103	1.0 (0.7–1.3)
	Hospital Admission	56 (54%)	-	-
	Nights in hospital	-	300	2.9 (1.4–4.4)
	……Admission through A&E	-	129	1.2 (0.6–1.8)
	……Direct admission	-	171	1.6 (0.3–3.0)
1 Year	Diagnostic Testing Status	63 (61%)	N/A	N/A
	ECG	50 (48%)	112	1.1 (0.7–1.4)
	EEG	41 (39%)	93	0.9 (0.6–1.2)
	CT	31 (30%)	58	0.6 (0.4–0.8)
	MRI	37 (36%)	69	0.7 (0.4–0.9)
	Other Tests	24 (23%)	59	0.6 (0.3–0.8)

This chart shows what portion of all respondents reported using a particular healthcare resource in a given recall period and, of those respondent, the frequency of healthcare utilization

Per capita and total annualized healthcare costs are shown in Table 3. Direct healthcare costs due to depression or anxiety averaged S$10,250 (95% CI: 7,150–13,350) per child. ED visits, hospitalizations and diagnostic tests accounted for 43% of these costs. At the population level, direct healthcare costs for youth with these conditions were estimated to be S$1.2bn (95% CI: S$1.1bn – S$1.4bn).

**Table 3:** Average per capita and total annual costs in Singapore dollars

Cost Category	Per Capita Cost, mean (95% CI)
***Direct Healthcare Costs***	***S$10,250 (S$7,150 – S$13,350)***
Medications	S$280 (S$230 – S$330)
In-Person Visits	S$3,380 (S$1,300 – S$5,450)
Televisits	S$2,150 (S$1,380 – S$2,920)
Diagnostic Tests	S$2,670 (S$920 – S$2,630)
ED Visit and Hospitalization Costs	S$1,780 (S$1,910 – S$3,430)
Total Economic Burden Costs, bn	S$1.223 (S$1.084 – S$1.385)

*Population estimates for number of children residents / citizens aged 4 to 21 years were sourced from SingStat (singstat.gov.sg).

ED: emergency department; CI: confidence interval

### School absenteeism and performance

A detailed breakdown of absenteeism and school performance is shown in Table 4. On average, youth with symptoms consistent with depression or anxiety missed 190 (95% CI: 126–254) hours of school (equivalent to approximately 24 days) because of their depression and anxiety symptoms in the past year. In total, 39% of youth missed the equivalent of one month of school or more and 13% missed the equivalent of three months or more. Parents reported an average school performance score of 6.3 (95% CI: 5.8–6.8), which means that school performance was reduced by 63% compared to what would have been expected had these youths not had depression and anxiety symptoms. Similarly, parents reported an average daily activities performance score of 5.8 (95% CI: 5.3–6.3), which is equivalent to a 58% reduction in these youths’ abilities to do their regular daily activities compared to their expected level if they had not had depression and anxiety symptoms.

**Table 4:** Child absenteeism and school performance (N = 104)

Productivity Loss Category	Mean (95% CI)
**Child Absenteeism Due to Depression or Anxiety**	
Hours Missed	190 (126–254)
% Missing > 1 Week (> 40 h)	57.7%
% Missing > 1 Month (> 160 h)	39.4%
% Missing > 3 Month (> 480 h)	12.5%
**Reduction in Performance Due to Mental Health Conditions**	
School Performance	62.9% (58.0 − 67.8%)
Daily Activities Performance	58.0% (52.8 − 63.2%)

*One month was defined as missing more than 160 h of school in a given month

## Discussion

This is the first study in Singapore, and among the first internationally, to estimate the prevalence and economic burden of depression and anxiety among youth using a low cost web panel approach with proxy reports from parents. The finding that 16.2% of parents reported symptoms consistent with depression or anxiety among their children and the corresponding healthcare burden and reductions in school and daily activities performance is cause for concern.

To contextualize our prevalence estimates, we compared them to the 2015 estimates of Singaporean youth ages 8 to 12 reported by Magiati et al. based on a household survey and more recent estimates from other countries. Magiati et al. reported estimates of depression and anxiety of 16.9% and 9.3%, respectively [12]. Using the web panel, parent report, and the PHQ-4 screening criteria, we found that 16.2% of youth ages 4 to 21 had symptoms consistent with these conditions. Given the divergent methodologies a direct statistical comparison is not advisable nor is it possible to tease out the influence of COVID-19 or other factors. However, for several reasons we suspect our prevalence estimate, and therefore burden estimates, are conservative. Not only are our estimates similar to results from 2015, they are lower than those of a recent systematic review of studies post onset of COVID-19. The review found estimates of these conditions ranging from 20.5 to 25.2% among youth aged 18 years or younger [11]. The most likely reason that our estimates are lower is underreporting in our sample due to reliance on proxy responses. Proxies may not be aware of the symptoms in some cases, may have difficulty recalling in others, or may not wish to disclose [36]. Lack of awareness, recall bias and systematic underreporting could be exacerbated in Singapore due to the high degree of stigma associated with these conditions [37]. To this point, our results were similar to those reported by Peng et al., where 16.3% (95% CI: 16.0 − 16.7%) and 10.3% (95% CI: 10.0 − 10.6%) of the high school students surveyed had symptoms of depression and anxiety respectively [21]. The study was conducted in Guangdong China in April 2020, after students had been attending compulsory home-based distance learning and lockdowns, similarly experienced by youths in Singapore, and where stigma from mental illness is also likely to be high.

Despite the high level of interaction with the health system, 84.8% of children with symptoms consistent with depression or anxiety did not have a formal diagnosis from a healthcare provider. This may result from multiple factors. First may be that those who screen positive do not actually have symptoms that warrant a clinical diagnosis. It is also possible that many of these youth were seen by a healthcare provider who recognized the conditions but did not codify a diagnosis to avoid stigma or because they felt the conditions may be temporary. It is also possible that some parents were told of a mental health diagnosis but did not recall that information or did not wish to disclose it. An alternative, and more concerning, possibility is that many youths with mental health conditions in Singapore remain untreated or undertreated. More research is needed to confirm this but, if confirmed, it represents a significant opportunity to improve mental healthcare as those with a formal diagnosis are most likely to receive effective treatment.

The children whose parents reported evidence of depression and anxiety symptoms incurred significant medical expenses. This may be because the most severe cases are more likely to be reported. Results suggest that of the over $10,000 in annual expenses per child associated with these conditions, 47% are potentially avoidable. This includes ED visits, hospitalizations and expensive differential diagnostic tests. The high utilization of expensive physiological diagnostic tests among youth may be indicative of a cautionary practice for pediatric populations to eliminate all differential diagnoses prior to the diagnosis or treatment of mental illnesses [38]. Further research is necessary to determine whether routine screening to identify and treat youth with mental health conditions, as suggested by the US Preventive Services Task Force in draft recommendations, would be cost-effective and potentially even cost saving in Singapore [39].

Addressing mental health among youth requires a whole-of-government approach. Coordinated efforts across ministries is required to offer appropriate severity-based services. In addition to implementing screening and personalized treatment programs, the government could also ramp up programs aimed to reduce stigma of mental health conditions among youth. Examples of such services include the Response, Early Intervention and Assessment in Community Mental Health (REACH) program, and the Community Health Assessment Team (CHAT), which provide community-centric personalized services to children and youth and promote peer support through a multi-sectoral approach [40–42]. They provide low-barrier access to mental health services in a stigma-free community setting. Other programs, such as “Beyond the Label” and “Nurture SG” also aim to increase awareness, reduce stigma, and promote access to care. Quantifying the cost-effectiveness of key components of such strategies should be an area of future research.

### Strengths and limitations

This study has both strengths and limitations. The primary strength is the ability to generate estimates of prevalence and burden of depression and anxiety using a low cost and expedient approach that takes advantage of an existing web panel. For the chosen panel, participants are recruited country wide to take surveys on a regular basis in exchange for modest rewards. Most households remain on the panel for 2–3 years. The panel exceeds 500,000 individuals and is broadly representative of the socioeconomic, gender, and ethnicity distributions in Singapore. However, a primary limitation is that it is unclear whether their children are representative of the general population of children in Singapore overall or among the subset with mental health conditions. It is possible that parents are more likely to report the more severe cases. If so our burden estimates may be inflated. In addition, whereas PHQ-4, as the combination of the PHQ-2 and GAD-2, is a validated instrument, it is a screening tool, not a diagnostic, tool. It allows for rapid identification of individuals who have symptoms consistent with these conditions but is not confirmatory. Our burden estimates are also based on self-report and could suffer from recall or other biases. Finally, although our prevalence estimates are based on all household members, our utilization and burden estimates are limited to the oldest child with symptoms of depression or anxiety in the household whose responding parent did not also report these symptoms. If these youth are not representative of all youth with these conditions, then our estimates would be biased. Future studies should aim to improve on our results using more robust school-based samples, face-to-face survey administration directly with youth, clinical diagnostic tools, and linkages to actual healthcare utilization and claims data.

## Conclusion

Even with significant potential for underreporting, these results reveal concerning rates of Singaporean youth with symptoms consistent with depression and anxiety, many of whom remain untreated. Results also reveal the short-term economic burden caused by these symptoms and hint at longer-term consequences resulting from poor school performance. This study should represent a call to action for Singapore to address poor mental health among youth.

## Electronic supplementary material

Below is the link to the electronic supplementary material.

Supplementary Material 1

## Data Availability

De-identified data will be made available to any group with an approved IRB upon reasonable request to the authors.

## Footnotes

1 1 S$ = 0.75 USD on 31st December 2022.
