# Supplementary material for: Healthcare utilization and costs of singaporean youth with symptoms of depression and anxiety: results from a 2022 web panel
Source: Child Adolesc Psychiatry Ment Health. 2023 May 11;17:60. doi: 10.1186/s13034-023-00604-z (PMC10173927; doi:10.1186/s13034-023-00604-z)
Supplement: Supplementary file 1 — Supplementary Material 1 [file 13034_2023_604_MOESM1_ESM.docx]

**Supplementary Materials Overview**

*Table of Contents*

Appendix A. Screening Questionnaire …………………………………………………..………..…………….Page 2

Appendix B. Child Respondent Survey …………………………………………………………………………Page 5

Appendix C. Data Quality Check and Cleaning……………………………………………………….……….Page 12

Supplementary Table S1. Unsubsidized Unit Cost Estimates for Healthcare Resources in Singapore……..…Page 13

**Appendix A. Main Respondent Survey**

*SCREENER PART 1: GENERAL QUESTIONS*

1. Are you a Singaporean Citizen or Permanent Resident (PR)?

| A | Yes |
| --- | --- |
| B | No |

[TERMINATE IF NO]

1. What is your current age?

|  |
| --- |

[TERMINATE IF <21]

1. How many children live in your household between ages 4 and 21?

| 1 | Zero |
| --- | --- |
| 2 | One |
| 3 | Two |
| 4 | Three |
| 5 | Four |
| 6 | Five |
| 7 | Six |
| 8 | Seven |
| 9 | Eight |
| 10 | More than eight |

[SKIP IF CODED 1 in 3]

1. Please enter the ages of all children between ages 4 and 21 living in your household from oldest age to youngest age:

|  | Enter number between 0 and 21 |
| --- | --- |
| Child #1 |  |
| Child #2 |  |
| Child #3 |  |
| Child #4 |  |

1. How many adult family members (>21 years of age) live in your household including yourself?

*[P/N: When directing to the proxy adult questionnaire and determining how many adults may be eligible, subtract one from the answer that’s selected]*

| 1 | Zero |
| --- | --- |
| 2 | One |
| 3 | Two |
| 4 | Three |
| 5 | Four |
| 6 | Five |
| 7 | Six |
| 8 | Seven |
| 9 | Eight |
| 10 | More than eight |

1. Please enter the ages of all adult family members living in your household from oldest age to youngest age.

*[P/N: When directing to the proxy adult questionnaire and determining how many adults may be eligible, subtract one from the answer that’s selected]*

|  | Must be >21 years of age |
| --- | --- |
| Adult #1 |  |
| Adult #2 |  |
| Adult #3 |  |
| Adult #4 |  |

1. Have any of the following individuals EVER been told by a physician that you have depression and/or an anxiety disorder?

|  | Yes, Depression | Yes, Anxiety Disorder | Yes, both | No |
| --- | --- | --- | --- | --- |
| Yourself | A | B | C | D |
| [Pipe in from 2a_Child]* | A | B | C | D |
| [Pipe in from 2a_Child]* | A | B | C | D |
| [Pipe in from 2a_AdultProxy]* | A | B | C | D |
| [Pipe in from 2a_AdultProxy]* | A | B | C | D |

*SCREENER PART 2: PHQ-4 Screener*

1. Over the last two weeks, how often have the following individuals been bothered by feeling nervous, anxious, or on edge?

|  | Not At all | Several Days | More than half the days | Nearly every day |
| --- | --- | --- | --- | --- |
| Yourself | 0 | 1 | 2 | 3 |
| [Pipe in from 2a_Child]* | 0 | 1 | 2 | 3 |
| [Pipe in from 2a_Child]* | 0 | 1 | 2 | 3 |
| [Pipe in from 2a_AdultProxy]* | 0 | 1 | 2 | 3 |
| [Pipe in from 2a_AdultProxy]* | 0 | 1 | 2 | 3 |

[**to pipe in based on number selected in 2a_Child & 2a_AdultProxy]*

1. Over the last two weeks, how often have the following individuals been bothered by not being able to stop or control worrying?

|  | Not At all | Several Days | More than half the days | Nearly every day |
| --- | --- | --- | --- | --- |
| Yourself | 0 | 1 | 2 | 3 |
| [Pipe in from 2a_Child]* | 0 | 1 | 2 | 3 |
| [Pipe in from 2a_Child]* | 0 | 1 | 2 | 3 |
| [Pipe in from 2a_AdultProxy]* | 0 | 1 | 2 | 3 |
| [Pipe in from 2a_AdultProxy]* | 0 | 1 | 2 | 3 |

[**to pipe in based on number selected in 2a_Child & 2a_AdultProxy]*

1. Over the last two weeks, how often have the following individuals been bothered by little interest or pleasure in doing things?

|  | Not At all | Several Days | More than half the days | Nearly every day |
| --- | --- | --- | --- | --- |
| Yourself | 0 | 1 | 2 | 3 |
| [Pipe in from 2a_Child]* | 0 | 1 | 2 | 3 |
| [Pipe in from 2a_Child]* | 0 | 1 | 2 | 3 |
| [Pipe in from 2a_AdultProxy]* | 0 | 1 | 2 | 3 |
| [Pipe in from 2a_AdultProxy]* | 0 | 1 | 2 | 3 |

[**to pipe in based on number selected in 2a_Child & 2a_AdultProxy]*

1. Over the last two weeks, how often have the following individuals been bothered by feeling down, depressed, or hopeless?

|  | Not At all | Several Days | More than half the days | Nearly every day |
| --- | --- | --- | --- | --- |
| Yourself | 0 | 1 | 2 | 3 |
| [Pipe in from 2a_Child]* | 0 | 1 | 2 | 3 |
| [Pipe in from 2a_Child]* | 0 | 1 | 2 | 3 |
| [Pipe in from 2a_AdultProxy]* | 0 | 1 | 2 | 3 |
| [Pipe in from 2a_AdultProxy]* | 0 | 1 | 2 | 3 |

[**to pipe in based on number selected in 2a_Child & 2a_AdultProxy]*

[Criteria for Inclusion in the Study]

- [If the participant scores >3 when summing Q8 and Q9] OR
- [If the participant scores >3 when summing Q10 and Q11]

Qualification Prioritization:

1. Main Respondent
2. Child (if more than 1 child, oldest child takes priority)
3. Proxy Adult (if more than 1 adult, the oldest adult takes priority)

**Appendix B. Child Survey.**

Thank you for completing the screener. Your responses indicate that you are eligible to participate in our online survey based on having at least one child experiencing symptoms of depression and/or anxiety. The following questionnaire asks you about their symptoms and it’s effects on school, work and healthcare use. If you have more than one child with the above symptoms, please ONLY consider the oldest among them when answer questions about “your child” throughout the survey. Please answer the questions to the best of your knowledge.

MAIN SURVEY – MEDICATION HISTORY

In the next section, you will be asked a series of questions on medications that your child may have taken or is currently taking to manage his or her depression/anxiety. We recommend that you have close access to your child’s medications and pill bottles to ensure more accurate responses.

1. Has your child **EVER** taken any prescription medications to treat his or her depression/anxiety?

| A | Yes, for Depression |
| --- | --- |
| B | Yes, for Anxiety |
| C | Yes, for Both |
| D | No |

[If Q1 = D, SKIP TO Q4]

1. In the **LAST 3 MONTHS**, has your child taken any prescription medications to treat his or her depression/anxiety?

| A | Yes, for Depression |
| --- | --- |
| B | Yes, for Anxiety |
| C | Yes, for Both |
| D | No |

[Q1 and Q2 in this section should show up on the same page]

[If Q2 = D, SKIP TO Q4]

1. Are you taking any of the following medications? Please check all that apply.

| A | Daily antidepressant (e.g., Prozac, Lexapro) |
| --- | --- |
| B | As needed anti-depressant or anti-anxiety drug (e.g., Xanax, Klonopin) |
| C | As needed sleep medicines to help with insomnia |
| D | Other (please specify): __________ |

MAIN SURVEY – HEALTHCARE UTILIZATION

1. In the **LAST 12 MONTH**S, has your child consulted a physician or other healthcare professional regarding his or her depression/anxiety?

| A | Yes |
| --- | --- |
| B | No |

[If Q4 = B, SKIP TO Q6]

1. In the LAST 3 MONTHS, has your child visited (including tele-visits) any of the following healthcare providers in an outpatient setting *because of his or her depression/anxiety*? If yes, please enter the number of visits. For visit types that have more than one healthcare provider listed, select all that apply.

| Visit Type | Yes | No | If yes, state the # of **in-person visits** in the LAST THREE MONTHS. Leave blank if not applicable. | If yes, state the # of **tele-visits** in the LAST THREE MONTHS. Leave blank if not applicable. | Please list whether **the last visit** was “public” or “private”? |
| --- | --- | --- | --- | --- | --- |
| Polyclinic |  |  |  |  |  |
| Private General Practitioner |  |  |  |  |  |
| Psychiatrist |  |  |  |  |  |
| Psychologist |  |  |  |  |  |
| Social Worker |  |  |  |  |  |
| Life Coach |  |  |  |  |  |
| Other |  |  |  |  |  |

[If “Yes” to “Polyclinic”, automatically note that the visit was “public”]

[If “Yes” to “Private General Practitioner”, automatically note that the visit was “private”]

1. In the LAST 12 MONTHS, has your child used any of the following services because of his or her depression/anxiety?

| Visit Type | Yes | No | Specify |
| --- | --- | --- | --- |
| Emergency department visit **without** hospital admission |  |  | How many in the last 12 months? |
| Emergency department visit **with** hospital admission |  |  | How many total nights did you spend including all admissions in the last 12 months? |
| Direct hospital admission **without** an emergency department visit |  |  | How many total nights did you spend including all admissions in the last 12 months? |

1. In the LAST 3 MONTHS, has your child used any of the following approaches to manage or treat his or her depression/anxiety?

| Therapy | Yes | No | If yes, please estimate the amount of money that you’ve spent on this therapy **in the last three months** in Singapore Dollars. |
| --- | --- | --- | --- |
| Acupuncture |  |  |  |
| Reflexology |  |  |  |
| Chiropractor Services |  |  |  |
| Professional Meditation Services |  |  |  |
| Other | Please specify: |  |  |

[If the respondent answers the amount of money spent question with a value >1,000 SGD, please provide a prompt to the respondent stating, “Can you please confirm that you spent more than 1,000 SGD in the last three months for these therapies?”]

1. In the LAST 12 MONTHS, has your child undergone any of the following medical tests due to his or her depression/anxiety? *Please type in the response in the text box to automatically select that option.*

| Test Type | Yes | No | Please list the total number of tests taken in the last 12 months. |
| --- | --- | --- | --- |
| Electrocardiogram (EKG) |  |  |  |
| Electroencephalogram (EEG) |  |  |  |
| CT/CAT Imaging |  |  |  |
| MRI Imaging |  |  |  |
| Other (Please Specify): |  |  |  |

MAIN SURVEY – ABSENTEEISM AND PRESENTEEISM (WPAI)

The following questions ask about the effect of your child’s mental health-related symptoms on their ability to work over the LAST 4 WEEKS, not including today. Please fill in the blanks or select a number, as indicated. Please only consider depression and/or anxiety symptoms.

1. Did your child miss school due to their symptoms in the LAST FOUR WEEKS?

| A | Yes |
| --- | --- |
| B | No |

[If answer to Q1 is NO, SKIP to Q3.]

1. During the LAST 4 WEEKS, how many hours did your child miss from school **because of problems associated with their symptoms of depression and/or anxiety**? *Include hours missed on days that your child did not work at all, days your child went in late, days your child left early, etc. because of his or her symptoms. Do not include time you missed to participate in this study.*

____________Hours

1. During the LAST FOUR WEEKS, how much did your child’s depression/anxiety symptoms affect **his or her school performance**? *If your child’s symptoms didn’t affect their school performance substantially, choose a low number. Choose a high number if your child’s symptoms affected their school performance.*

| No symptoms and/or symptoms had no effect on my work | 0 | 1 | 2 | 3 | 4 | 5 | 6 | 7 | 8 | 9 | 10 | Symptoms completely prevented me from working |
| --- | --- | --- | --- | --- | --- | --- | --- | --- | --- | --- | --- | --- |

1. During the LAST FOUR WEEKS, how much did your child’s depression/anxiety symptoms **affect their ability to do his or her regular daily activities, other than school performance**? *By regular activities, we mean the usual activities your child does such as work around the house, playing sports, making artwork, playing with other kids. If your child’s symptoms affected his or her activities only a little, choose a low number. Choose a high number if your child’s symptoms affected their activities a great deal.*

| No symptoms and/or symptoms had no effect on his or her regular daily activities | 0 | 1 | 2 | 3 | 4 | 5 | 6 | 7 | 8 | 9 | 10 | Symptoms completely prevented him or her from his or her regular daily activities |
| --- | --- | --- | --- | --- | --- | --- | --- | --- | --- | --- | --- | --- |

1. When your child stayed home due to their symptoms, who cared for them? Please check all that apply.

| A | You |
| --- | --- |
| B | Your Spouse or Co-Parent |
| C | Other Family Member or Friend |
| D | Helper/Paid Caregiver |

The following questions ask about the effect of your child’s symptoms on their unpaid caregiver’s (you, your spouse, other family member or friend) work in the LAST 4 WEEKS. When answering, please ONLY consider the individual who provided the most care for your child when they were home due to their symptoms. This individual will be simply referred to as “caregiver” in the upcoming questions.

1. Is this caregiver currently employed or self-employed (i.e., working for pay)?

| A | Yes |
| --- | --- |
| B | No |

1. During the LAST 4 WEEKS, how many days did the caregiver work?

____________Days

1. During the LAST 4 WEEKS, how many hours did the caregiver miss from work because of problems associated with your child’s symptoms of depression and/or anxiety? *Include hours missed on days you did not work at all, days you went in late, days you left early, etc. because you were caring for your child (e.g., accompanies them to their doctor appointments, visited hospitals or clinics, or helped them dress, groom, eat, or take medications). Do not include time you missed to participate in this study.*

____________Hours

9. What is this caregiver’s typical personal monthly employment income from all sources?

| 1 | Less than $1000 |
| --- | --- |
| 2 | $1000 - $1999 |
| 3 | $2000 - $2999 |
| 4 | $3000 - $3999 |
| 5 | $4000 - $4999 |
| 6 | $5000 - $5999 |
| 7 | $6000 - $6999 |
| 8 | $7000 -$9999 |
| 9 | $10,000 and over |
| 99 | Prefer not to answer |

MAIN SURVEY – QUALITY OF LIFE ASSESSMENT (EQ5D-5L)

Under each heading, please tick the ONE box that best describes your child’s health TODAY.

Mobility

|  | My child has no problems in walking about |
| --- | --- |
|  | My child has slight problems in walking about |
|  | My child has moderate problems in walking about |
|  | My child has severe problems in walking about |
|  | My child is unable to walk about |

Self-Care

|  | My child has no problems washing or dressing himself or herself |
| --- | --- |
|  | My child has slight problems washing or dressing himself or herself |
|  | My child has moderate problems washing or dressing himself or herself |
|  | My child has severe problems washing or dressing himself or herself |
|  | My child is unable to wash or dress himself or herself |

Usual Activities (e.g., work, study, housework, family or leisure activities)

|  | My child has no problems doing his or her usual activities |
| --- | --- |
|  | My child has slight problems doing his or her usual activities |
|  | My child has moderate problems doing his or her usual activities |
|  | My child has severe problems doing his or her usual activities |
|  | My child is unable to do his or her usual activities |

Pain/Discomfort

|  | My child has no pain or discomfort |
| --- | --- |
|  | My child has slight pain or discomfort |
|  | My child has moderate pain or discomfort |
|  | My child has severe pain or discomfort |
|  | My child has extreme pain or discomfort |

Anxiety/Depression

|  | My child is not anxious or depressed |
| --- | --- |
|  | My child is slightly anxious or depressed |
|  | My child is moderately anxious or depressed |
|  | My child is severely anxious or depressed |
|  | My child is extremely anxious or depressed |

-
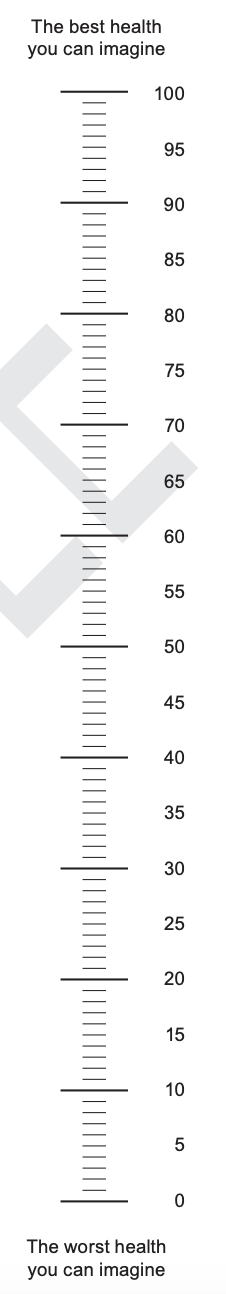
We would like to know how good or bad your child’s health is TODAY.
- This scale is numbered from 0 to 100.
- 100 means the best health you can imagine. 0 means the worst health you can imagine.
- Mark an X on the scale to indicate how your child’s health is TODAY.
- Now, please write the number you marked on the scale here: _______

**Appendix C. Data Quality Check and Cleaning**

Data was first thoroughly checked for straight line answers. No respondents provided straight line answers for questions pertaining to medication use or healthcare resource utilization (i.e., indicating that they had used all healthcare resources for the same number of times in the recall period). Data was next checked for skip logic. Seventeen respondents reported some form of healthcare resource utilization (e.g., in-person visits, tele-visits, hospital resources, and diagnostic tests) despite responding to a prior question that they did not use any resources. All of these responses were recoded as missing for each respective sub-section. Thirty-eight respondents reported that their child missed school hours despite recording in a previous question that they did not miss school. All of these responses were recorded as missing for each respective sub-section.

**Supplementary Table 1. Unsubsidized unit cost estimates of an demand for healthcare resources in Singapore.**

*Retrieved between June 2022 and August 2022. All costs include Goods and Services Tax (GST) of 7%.*

| Category | Unit Cost (SGD) | | Details | Sources |
| --- | --- | --- | --- | --- |
| Medications |  |  | |  |
| Daily Anti-Depressant or Daily Anti-Anxiety | $ 19.20 | Average monthly cost of the most common daily anti-depressant and/or anti-anxiety. | | Institute for Mental Health |
| As Needed Anti-Depressant or Anti-Anxiety | $ 24.00 | Average monthly cost of maintenance dose anti-depressant or anti-anxiety drug. Assume 50% utilization. | | Institute for Mental Health |
| As Needed for Insomnia | $ 24.00 | Average monthly cost of maintenance dose anti-depressant or anti-anxiety drug. Assume 25% utilization. | | Institute for Mental Health |
| Other Medication | $ 19.20 | Average monthly cost of the most common daily anti-depressant and/or anti-anxiety. | | Institute for Mental Health |
| Medication Not Listed Above | $ 19.20 | Average monthly cost of the most common daily anti-depressant and/or anti-anxiety. | | Institute for Mental Health |
| Physician Visits and Outpatient Procedures |  |  | |  |
| Polyclinic | $ 53.50 | Average of non-resident prices for General Practitioner consultations. | | Online payment schedules for SingHealth Polyclinics, National Healthcare Group, and National University Polyclinics |
| Private General Practitioner | $ 45.20 | Average of non-resident prices for Private General Practitioner consultations. | | Online payment schedules for Prohealth 24-hour, Central 24-hour Clinic (Woodlands), Thomson 24-Hr Family Clinic, and Intermedical 24-Hr Clinic |
| Psychiatrist - Public (Non-Subsidised) | $ 102.25 | Average of non-subsidized prices for initial and subsequent consultations for an adult at IMH. | | Institute for Mental Health |
| Psychologist - Private | $ 237.40 | Average of psychologist consultation fees (private practice, non-subsidized). | | Online payment schedules for Raffles Counselling Centre and Alliance Counseling Center |
| Social Worker | $ 95.00 | Average cost of a single one-hour counseling session with a social worker at IMH. | | Institute for Mental Health |
| Life Coach | $ 218.00 | Average of online session costs for career counseling. | | Online payment schedules for American Association of Singapore, Gary and Pearl International, Anagram Group, and Emunah Coaching |
| Diagnostic Tests |  |  | |  |
| EKG | $ 21.40 | Average cost of an EKG in Singapore. | | 1. Institute for Mental Health Singapore |
| EEG | $ 200.00 | Average cost of a neurology consult which would include an EEG. | | 1. Singapore General Hospital |
| CT/CAT Scan | $ 1,350.00 | Average price per CT/CAT scan at a public hospital. | | 1. Singapore General Hospital |
| MRI Imaging | $ 1,850.00 | Average price per MRI scan at a public hospital. | | 1. Singapore General Hospital |
| Emergency Department Visit | $ 127.00 | Average cost of an ED visit to IMH. | | 1. Institute for Mental Health Singapore |
| Hospitalization Costs Per Day | $ 555.00 | Average per daily ward and treatment fee for a one-bedded ward. | | 1. Institute for Mental Health Singapore |
